# CircRNA_100290 promotes GC cell proliferation and invasion via the miR-29b-3p/ITGA11 axis and is regulated by EIF4A3

**DOI:** 10.1186/s12935-021-01964-2

**Published:** 2021-06-28

**Authors:** Gang Wang, Dan Sun, Wenhui Li, Yan Xin

**Affiliations:** 1 Laboratory of Gastrointestinal Onco-Pathology, Cancer Institute, The First Affiliated Hospital of China Medical University, 155 Nanjing North Street, Heping District, Shenyang, Liaoning Province China; 2 Department of Oncology, The First Affiliated Hospital of Shandong First Medical University & Shandong Provincial Qianfoshan Hospital, Shandong Key Laboratory of Rheumatic Disease and Translational Medicine, Shandong Lung Cancer Institute, 16766 Jingshi Road, Lixia District, Jinan, Shandong Province China

**Keywords:** circRNA_100290, miR-29b-3p/ITGA11 axis, EIF4A3, EMT, Gastric cancer

## Abstract

**Background:**

Circular RNAs (circRNAs) have been reported to be important regulators of the development and progression of various carcinomas. However, the role of circRNA_100290 in gastric cancer (GC) is still unclear. This study aimed to investigate the role of circRNA_100290 in GC invasion and metastasis and the possible underlying mechanism.

**Methods:**

The expression of circRNA_100290 in GC cells and tissues was examined using quantitative real-time polymerase chain reaction (qRT-PCR). The role of circRNA_100290 in cell proliferation, migration, and invasion was evaluated in the AGS and HGC-27 cell lines in vitro. Bioinformatics tools, dual-luciferase reporter assays, Western blot assays and qRT-PCR were used to explore the pathways downstream of circRNA_100290. The mechanism underlying the regulation of circRNA_100290 expression was explored using RNA immunoprecipitation, qRT-PCR, and Western blot assays.

**Results:**

The expression of circRNA_100290 was significantly upregulated in GC cells and 102 GC tissues, and high circRNA_100290 expression in GC was closely related to Borrmann’s type, lymph node metastasis and tumour-node-metastasis stage. In vitro, knockdown of circRNA_100290 in AGS and HGC-27 cells significantly inhibited cell proliferation, migration, and invasion. Mechanistically, a dual-luciferase reporter assay confirmed the direct interaction between circRNA_100290 and miR-29b-3p, which targets ITGA11, an oncogene that is closely related to epithelial–mesenchymal transition (EMT). In addition, EIF4A3, an RNA-binding protein (RBP), could inhibit the formation of circRNA_100290 by binding to the flanking sites of circRNA_100290. Low EIF4A3 expression in GC was related to a poor prognosis.

**Conclusions:**

Elevated circRNA_100290 expression in GC promotes cell proliferation, invasion and EMT via the miR-29b-3p/ITGA11 axis and might be regulated by EIF4A3. CircRNA_100290 might be a promising biomarker and target for GC therapy.

**Graphical abstract:**

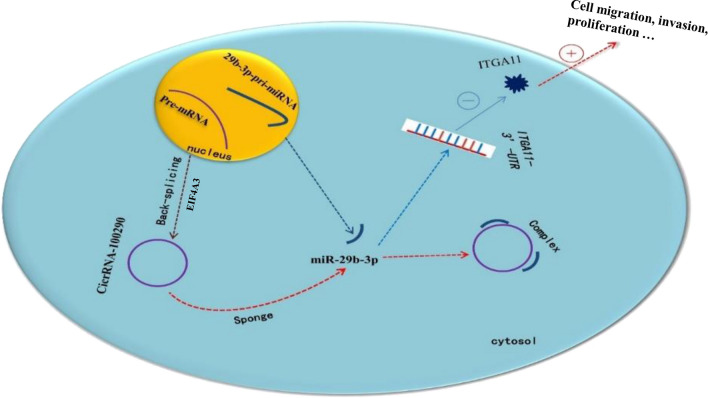

## Background

Gastric cancer (GC) is the fifth most frequently diagnosed malignancy and the third leading cause of cancer-related death worldwide [1]. According to Chinese cancer statistics, in 2014, the number of new cases of GC in China was approximately 410,000, and the number of GC-related deaths was approximately 294,000, which was second only to the number of lung cancer-related deaths [2]. Although many advances have been made in diagnostics and new therapeutic approaches for GC, a large number of patients with GC have a poor prognosis. Therefore, appropriate molecular biomarkers for early diagnosis and potential treatment targets for GC need to be identified.

CircRNAs are a unique class of RNA molecules formed via back-splicing. CircRNAs have neither 5–3’ polarities nor polyadenylated tails; circRNAs were first identified in plant viruses in the 1970s and are now known to be widely expressed in eukaryotes [3, 4]. Recently, various circRNAs have been found to participate in tumorigenesis and cancer progression [5, 6]. CircRNAs are often suitable biomarkers of diseases because they have covalently closed-loop structures, are more stable than the corresponding linear RNAs, and are resistant to degradation by RNase R. In addition, circRNAs often exhibit specific expression in various diseases and tissues [4, 7, 8]. More convincing evidence has demonstrated that circRNAs are dysregulated in GC. CircRNA_001569 and circPDSS1 were reported to be overexpressed in GC and to accelerate GC progression by sponging miR-145 and miR-186-5p, respectively [9, 10]. Circ_0027599/PHDLA1, circLARP4, and circPVRL3 function as tumour suppressors and inhibit the growth and metastasis of GC cells [11–13]. Previous studies reported that circRNA_100290 was upregulated and functioned as a miRNA sponge in oral squamous cell carcinoma and colorectal cancer [14, 15]. However, the role of circRNA_100290 in GC is still unclear. In addition, splicing factors and RNA-binding proteins (RBPs) might regulate the formation of circRNAs via back-splicing [16, 17], but the mechanism by which circRNA_100290 is regulated in GC is still unknown.

The present study was novel in demonstrating that circRNA_100290 was overexpressed in GC samples and cell lines. In vitro, silencing the expression of circRNA_100290 suppressed GC cell proliferation, induced G0/G1 phase arrest, and impeded migration, invasion, and EMT via the miR-29b-3p/ITGA11 axis. The present study provides a promising biomarker and an effective target for GC treatment.

## Methods

### Clinical specimen collection

A total of 102 fresh GC and matched normal gastric epithelial tissues (> 5 cm away from the tumour site) were acquired from patients with GC undergoing resection at The First Affiliated Hospital of China Medical University. All the clinicopathological data were classified according to the Eighth Edition of AJCC Clinical Practice Guidelines for GC. This study was approved by the ethics committee of The First Affiliated Hospital of China Medical University, and informed consent was obtained from the patients.

### Cell culture and transfection

Four GC cell lines, AGS, BGC-823, SGC-7901, and HGC-27, and the human immortalized normal gastric epithelial cell line GES-1 were provided and characterized by GeneChem Co., Ltd. (Shanghai, China). Hsa_circ_100290 and EIF4A3 siRNA and the corresponding negative control sequences were designed and synthesized by GenePharma (China). The si-circRNA_100290 sequence of the sense strand was 5’-CUCAUGCUUAGGCUUGAUUdTdT-3’; the sequence of the antisense strand was 3’-dTdTGAGUACGAAUCCGAACUAA-5’. The si-EIF4A3 sequence of the sense strand was 5’-CGAGCAAUCAAGCAGAUCAdTdT-3’, and the sequence of the antisense strand was 3’-dTdTGCUCGUUAGUUCGUCUAGUdTdT-5’. AGS and HGC-27 cells were prepared for si-circRNA_100290 and si-EIF4A3 transfection using Lipofectamine reagent (GenePharma, China) according to the manufacturer’s protocol. The knockdown efficiency was detected by quantitative real-time polymerase chain reaction (qRT-PCR) 48 h after transfection.

### RNA extraction and real-time PCR

RNA was extracted from GC cells and tissues using miRcute miRNA Kits (Tiangen Biotech Co., Ltd, Beijing, China) following the instructions. The concentration and quality of RNA were evaluated using a NanoDrop spectrophotometer (Thermo Scientific, USA). Then, the extracted mRNA and miRNA were subjected to reverse transcription using the PrimeScript Master Mix (TaKaRa, Japan) and MiR-X miRNA First-Strand Synthesis Kit (Takara, Japan), respectively, according to the manual’s instructions. The primers were designed and synthesized by Sangon Biotech (Shanghai, China). qRT-PCR assays were performed, and the expression levels were calculated using the 2- Δ Ct method and normalized to GAPDH. The reaction conditions for circRNA_100290 were as follows: 95 °C for 30 s; 40 cycles of 95 °C for 5 s, annealing at 56 °C for 32 s; dissolving curve at 95 °C for 15 s, 60 °C for 30 s, and 95 °C for 15 s. The primers for circRNA_100290 were as follows: 5’-CACGGACACAGTCATTCCCT-3’ and 5’ATCAAGCCTAAGCATGAGA ATGAAA-3’. The primers for miR-29b-3p were as follows: 5’-TAGCACCATTT GAAATCAGTGTT-3’. The primers for ITGA11 were as follows: 5’-GGAGGAAGACTTGCGTCG-3’ and '5’-CACAGGTTCCCCAGTAGATG-3’. The primers for EIF4A3 were as follows: 5’-CGCGGACTCTGACATATGGCGACCACGGCCACGATG-3’ and 5’-TCCCGCAGGCCCATGGTGTCG-3’.

### Plasmid transfection and luciferase assays

Luciferase reporter gene plasmids containing the wild-type 3’-UTR or mutated 3’-UTR of circRNA_100290 as well as the miR-29-3p overexpression plasmid were generated by GeneChem (GeneChem, China). Then, 20 ng reporter construct and 80 ng miRNA expression plasmid, along with 4 ng Renilla luciferase plasmid, were cotransfected into HEK 293 T cells in a 96-well plate using jetPRIME transfection reagent (PolyPlus, France) as described by the manufacturer. The transfection efficiency was evaluated by fluorescence microscopy. The luciferase activity was measured 48 h after transfection using the dual-luciferase reporter assay system as described by the manufacturer (Promega, USA).

### Western blot

Western blot assays were conducted following a previous report [18]. The following primary antibodies were used: anti-EIF4A3 (1:1000, Abcam, USA), anti-E-cadherin (1:1000, CST, USA), anti-Vimentin (1:1000, CST, USA), anti-N-cadherin (1:1000, CST, USA) and anti-GAPDH (1:1000, Origene Co., Ltd., Beijing, China).

### RNA immunoprecipitation assay

RNA-binding protein immunoprecipitation (Millipore, USA) was used to perform an RIP assay. According to the manufacturer’s protocol, 1 × 107 cells were harvested and lysed in complete RIPA lysis buffer. RNA magnetic beads were conjugated to anti-EIF4A3 (Abcam, USA) or control anti-IgG (Millipore, USA). The Ct value of circRNA_100290 was detected by qRT-PCR.

### Colony formation assay

Two hundred GC cells were seeded in six-well plates and incubated for 11 days. After being washed, fixed, and stained with 0.01% crystal violet, the cell colonies were imaged and evaluated using Quantity One software.

### Cell counting kit (CCK-8) assay

Two thousand GC cells were plated in 96-well plates. Ten microlitres of CCK-8 (Solarbio, Beijing, China) reagent was added and incubated for 3 h at 37 °C atmosphere. Then, the absorbance at 450 nm was detected and recorded for five consecutive days.

### Flow cytometry analysis for cell cycle

Cell fixation was conducted using 70% ethanol for 24 h. Then, the cells were stained using PI and RNase reagent and incubated for 30 min at 37 °C. Cell cycle distribution analysis was performed using a flow cytometry device (BD, USA).

### Wound healing assay

Approximately 3 × 10^5^ cells were plated into a 6-well dish. A linear scratch was made using a sterile 10-µL pipette tip when the cell confluence reached 90%. The cell scratch wound was washed with PBS and treated with RPMI 1640 supplemented with 3% foetal bovine serum. The cell scratch wound was imaged under a microscope after 0, 24, and 48 h. Then, the wound healing rate was analysed using ImageJ software.

### Transwell migration and invasion assay

Transwell inserts (Corning, USA) were used to perform cell migration and invasion assays. The membrane was not coated with Matrigel (BD, USA) for the cell migration assay. GC cells were added to the upper chamber and cultured in an incubator at 37 °C for 24 h. Then, the migrated cells were fixed, stained with crystal violet and recorded. For the cell invasion assay, the membrane was coated with Matrigel and serum-free medium mixture (BD, USA), and the culture was incubated at 37 °C for 48 h.

### Bioinformatics analysis

Bioinformatics prediction was performed using RNA22 v2.0 (https://cm.jefferson.edu/rna22/Interactive/), starBase (https://starbase.sysu.edu.cn/starbase2), and Circinteractome (https://omictools.com/circinteractome-tool) [19–21]. The data of the miRNA and mRNA arrays were obtained from The European Bioinformatics Institute (EBI, www.ebi.ac.uk/), Gene Expression Omnibus (GEO, https://www.ncbi.nlm.nih.gov/gds) and The Cancer Genome Atlas (TCGA, https://cancergenome.nih.gov/) [22–24]. The functional protein association of ITGA11 was analyzed using STRING (http://string-db.org/) [25]. The survival curve was plotted using R software and Kaplan–Meier plotter (www.kmplot.com/) [26].

### Statistical analysis

All the statistical analyses were conducted using SPSS 22.0 (IBM, NY, USA). The data are presented as the mean ± standard error of the mean. The χ2 test, Student’s t-test, and one-way analysis of variance were used for comparisons. The Pearson correlation coefficient was calculated to measure the correlation between factors. A P value less than 0.05 was considered statistically significant.

## Results

### Expression of CircRNA_100290 was upregulated in GC tissues

**Fig. 1:**
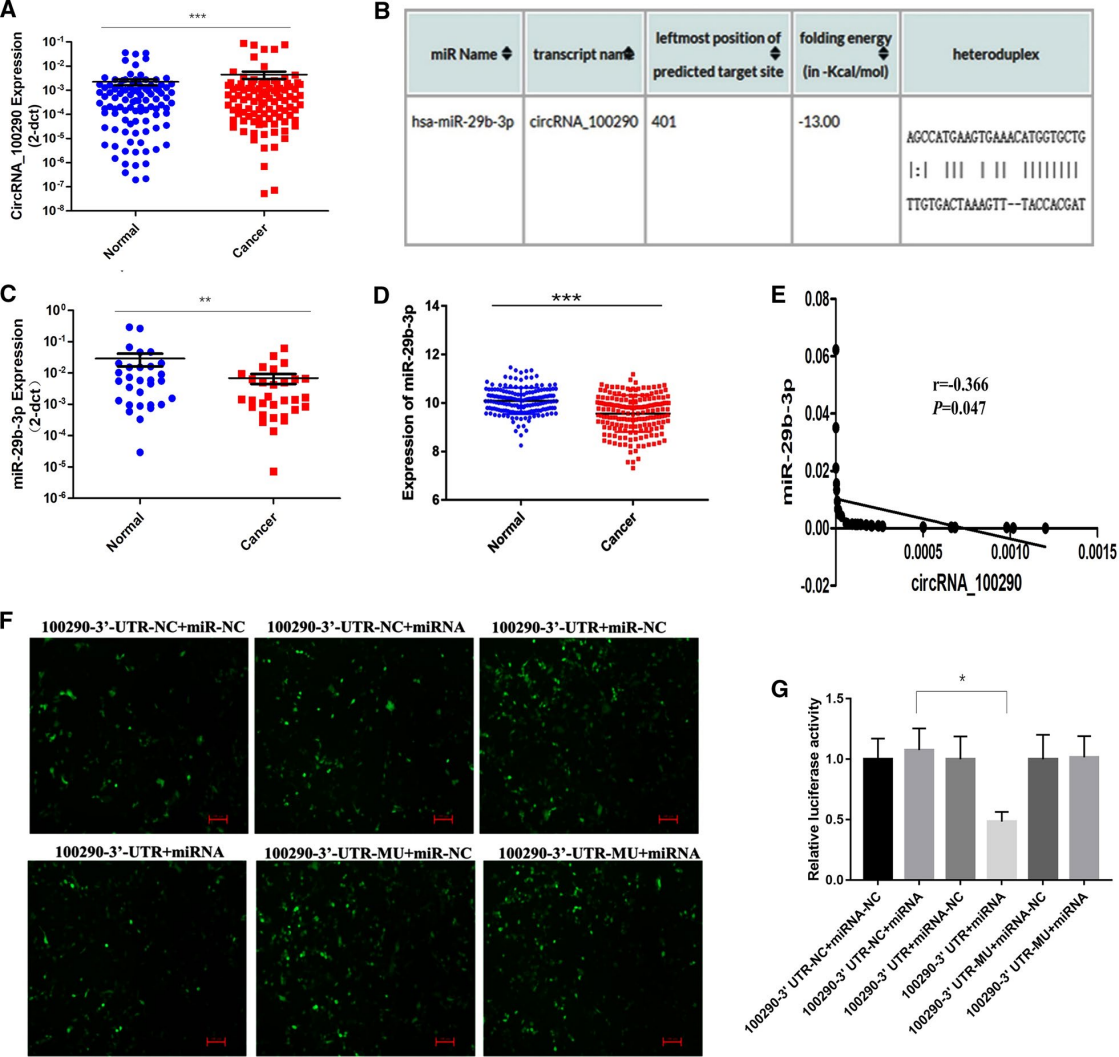
The expression patterns and correlation of circRNA_100290 and hsa-miR-29b-3p expression in human GC. **A** CircRNA_100290 expression in 102 GC tissues and paired normal tissues was detected by qRT-PCR. The data are presented as the mean ± SEM.****P < 0.0001. **B** *RNA22 V2.0* was used to predict the interaction between circRNA_100290 and hsa-miR-29b-3p. **C** The expression of miR-29b-3p in 31 GC tissues and matched normal mucosa tissues was evaluated by qRT-PCR. The data are presented as the mean ± SEM. **P < 0.01. **D** Expression analysis of miR-29b-3p in 184 GC tissues and 168 normal tissues was performed according to the EBI database. ***P < 0.001. **E **A negative correlation between circRNA_100290 and miR-29b-3p expression was observed by Pearson correlation analysis. r = − 0.366, P = 0.047. **F** Evaluation of the transfection efficiency of the luciferase reporter gene plasmid by fluorescence microscopy. **G** The relative luciferase activity of each group was calculated. The luciferase activity of the control group was normalized to 1, *P < 0.05

qRT-PCR was performed to examine the expression of circRNA_100290 in 102 paired GC tissues. The results showed that the expression of circRNA_100290 in GC tissues was significantly higher than that in paired adjacent noncancerous tissues (Fig. 1A). Moreover, analysis of clinicopathological characteristics revealed that the expression of circRNA_100290 in GC tissues was closely related to Borrmann’s type, lymph node metastasis and TNM stage (Table 1).

**Table 1:** Relevance analysis between expression of circRNA_100290 and clinicopathological characteristics in Chinese patients with GC

Characteristics	N = 102	Expression of circRNA_100290		χ2	*P*
		Low	High		
Gender				0.788	0.375
Male	74	35	39 (52.7%)		
Female	28	16	12 (35.7%)		
Age (years)				0.382	0.537
≤ 60	37	17	20 (54.1%)		
> 60	65	34	31 (47.7%)		
Borrmann’s types				4.320	**0.038**
I + II	13	10	3 (23.1%)		
III	89	41	48 (53.9%)		
WHO’s histological types				3.588	0.310
Tubular ade					
Moderately diff	29	16	13 (44.8%)		
Poorly diff	63	28	35 (55.6%)		
Mucinous ade	8	5	3 (37.5%)		
Signet ring cell car	2	2	0 (0.00%)		
Lauren’s types				4.738	0.094
Intestinal	22	15	7 (31.8%)		
Diffuse	70	33	37 (52.9%)		
Mixed	10	3	7 (70.0%)		
Ln metastasis				12.262	**0.002**
N0	24	19	5 (20.8%)		
N1 + N2	42	20	22 (52.4%)		
N3	36	12	24 (66.7%)		
Distant metastasis					0.118^a^
M0	98	51	47 (40.8%)		
M1	4	0	4 (100%)		
TNM Staging				8.513	**0.004**
I + II	27	20	7 (25.9%)		
III + IV	75	31	44 (58.7%)		

^a^ Calculated by a Fisher’s exact test

*Ade* Adenocarcinoma,* Diff* Differentiated,* Car* Carcinoma,* Ln* Lymph node

### Expression of miR-29b-3p decreased in GC and could be sponged by circRNA_100290

CircRNAs customarily function as miRNA sponges to bind functional miRNAs. Hence, RNA22 V2.0 was used to predict miRNAs with potential circRNA_100290 binding sites (Fig. 1B). Then, miR-29b-3p was chosen for further research. qRT-PCR assays were conducted to examine the expression of miR-29b-3p in 31 matched GC tissues. The results showed diminished expression of miR-29b-3p in GC tissues (Fig. 1C, P < 0.01). Subsequently, the expression of miR-29b-3p in GC was validated by analyzing data downloaded from the EBI database, which included 184 GC tissues and 168 normal gastric epithelium tissues, and the same expression trend was observed (Fig. 1D, P < 0.001). The correlation analysis results demonstrated a negative correlation between the expression of circRNA_100290 and miR-29b-3p in GC tissues (r = –0.3656, P = 0.047; Fig. 1E). Additionally, luciferase reporter plasmids were transfected into HEK 293 T cells to further explore the relationship between circRNA_100290 and miR-29b-3p. The dual-luciferase reporter assay revealed that miR-29b-3p mimics reduced the luciferase activity in the wild-type group, implicating miR-29b-3p as a target for circRNA_100290 (Fig. 1F, G). These data suggested that miR-29b-3p might act as a tumour suppressor and that circRNA_100290 could serve as a molecular sponge for miR-29b-3p in GC.

### Expression of ITGA11 was increased in GC and was negatively correlated with the expression of miR-29b-3p in GC tissues

**Fig. 2:**
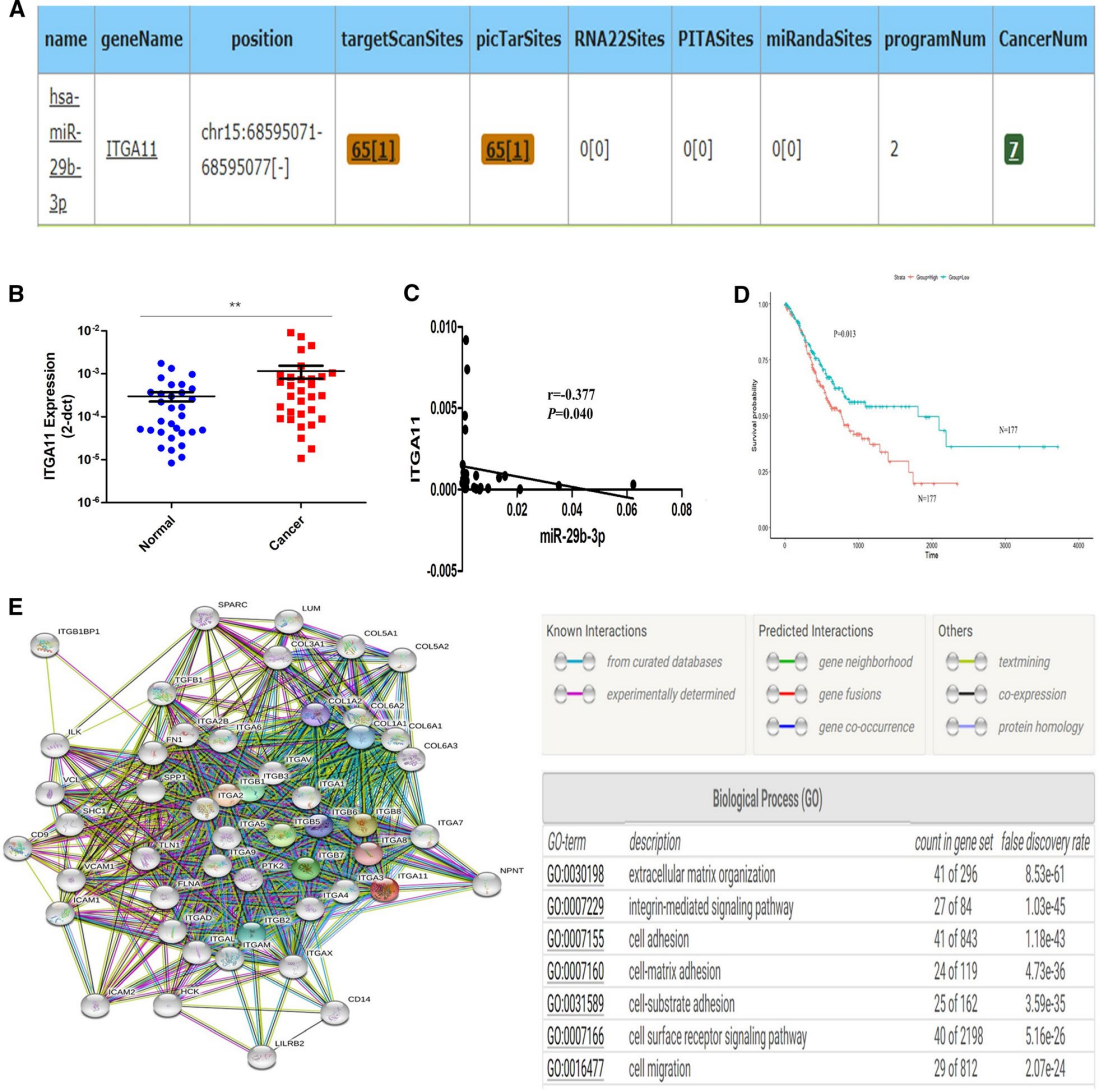
The correlation of hsa-miR-29b-3p and ITGA11 expression in human GC. **A** One of the most likely target genes of hsa-miR-29b-3p was predicted by starBase. The results showed that ITGA11 had binding sites with miR-29b-3p. **B** *ITGA11* expression in 31 GC tissues and paired normal tissues was detected by qRT-PCR. **P < 0.01. **C** A negative correlation between miR-29b-3p and *ITGA11* expression was observed by Pearson correlation analysis. r = − 0.317, P = 0.009. **D** Prognostic value of the expression of *ITGA11* in 354 patients with GC in the TCGA database. **E** Protein–protein interaction analysis and gene ontology analysis of ITGA11 using STRING software

StarBase was used to predict the possible target genes of miR-29b-3p. ITGA11, a potential target gene, might have binding sites in the 3’-UTR of miR-29b-3p (Fig. 2A). The expression and function of ITGA11 in GC are not completely clear. qRT-PCR was conducted to examine the expression of ITGA11 in 31 GC tissues. The results showed that the expression level of ITGA11 in GC tissues was higher than that in paired noncancerous tissues (Fig. 2B). Data were downloaded from the TCGA database to further explore the role ITGA11 plays in GC. Clinicopathological characteristic analysis showed that ITGA11 expression was closely related to Lauren’s type, invasion depth and TNM stage (Table 2). Furthermore, the correlation analysis showed a negative correlation between the expression of miR-29b-3p and ITGA11 in GC (r =  − 0.3773, P = 0.040; Fig. 2C).

**Table 2:** Relevance analysis between expression of ITGA11 and clinicopathological characteristics in GC patients from TCGA database

Characteristics	N	Expression of ITGA11		χ2	*P*
		Low	High		
Gender	375			2.161	0.142
Male	241	114	127 (52.7%)		
Female	134	74	60 (44.8%)		
Age (years)	371			0.61	0.435
< 65	155	81	74 (47.7%)		
≥ 65	216	104	112 (51.9%)		
Histological grade	366			1.921	0.166
G1 + G2	147	80	67 (45.6%)		
G3	219	103	116 (53.0%)		
Lauren’s types^a^	229			7.323	**0.026**
Intestinal	157	77	80 (51.0%)		
Diffuse	55	18	37 (67.3%)		
Mixed	17	4	13(76.5)		
Depth of invasion	367			22.669	**0.000**
T1	19	18	1 (5.30%)		
T2	80	46	34 (42.5%)		
T3	168	85	83 (49.4%)		
T4	100	38	62 (62.0%)		
Ln metastasis	357			5.471	0.14
N0	111	64	47 (42.3%)		
N1	97	52	45 (46.4%)		
N2	75	33	42 (56.0%)		
N3	74	32	42 (56.8%)		
Distant metastasis	355			1.169	0.28
M0	330	169	161 (48.8%)		
M1	25	10	15 (60.0%)		
TNM Staging	352			13.737	**0.003**
I	53	39	14 (26.4%)		
II	111	54	57 (51.4%)		
III	150	69	81 (54.0%)		
IV	38	16	22 (57.9%)		

*Ln* Lymph node

^a^Lauren’s types data of 146 GC cases is unavailable

Subsequently, the prognostic value of the expression of ITGA11 in 354 GC patients from the TCGA database was evaluated. The ITGA11 high-expression group exhibited lower 5-year survival rates (Fig. 2D, P = 0.013). High ITGA11 expression predicted poor prognosis in GC. In addition, protein–protein interaction (PPI) analysis of ITGA11 was conducted using STRING software. Potential proteins interacting with ITGA11 include integrin family members, collagen family members, transforming growth factor family members, and so on. Gene ontology (GO) analysis revealed that the aforementioned proteins were mainly involved in migration-related biological processes, such as extracellular matrix organization, cell adhesion and migration (Fig. 2E).

### CircRNA_100290 promoted the proliferation, colony formation, and cell cycle distribution of GC cells via the miR-29b-3p/ITGA11 axis

**Fig. 3:**
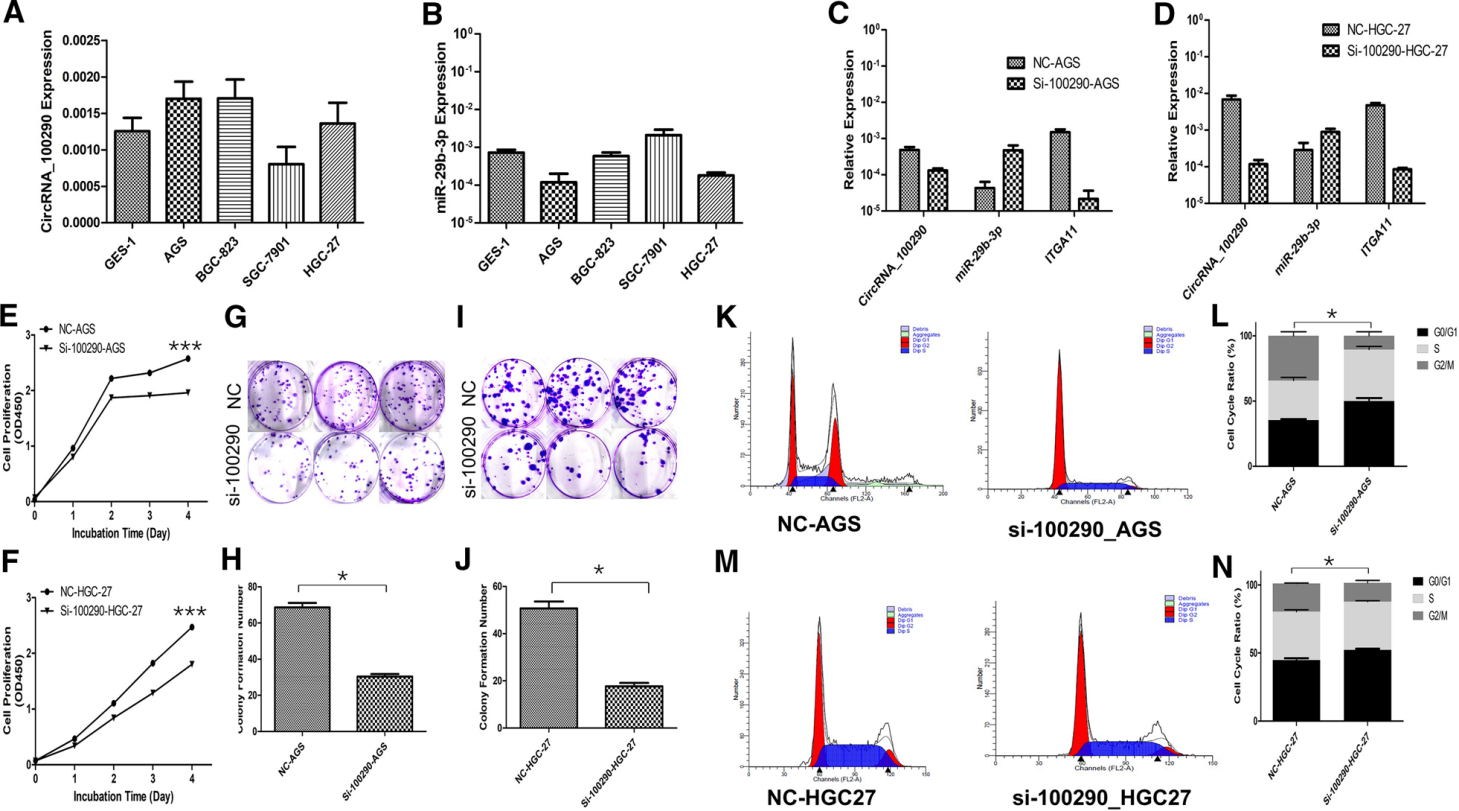
Knocking down circRNA_100290 inhibits GC cell viability and colony formation and induces G0/G1 cycle arrest. **A** Expression of circRNA_100290 was detected in four GC cell lines, AGS, BGC823, SGC7901, HGC27, and GES-1 human immortalized normal gastric epithelial cells by qRT-PCR assay. **B** Expression of miR-29b-3p was detected in the AGS, BGC823, SGC7901, HGC27 and GES-1 cell lines by qRT-PCR assay. **C** Detection of miR-29b-3p and ITGA11 expression in the AGS cell line by qRT-PCR assay after circRNA_100290 siRNA transfection was conducted. **D** Detection of miR-29b-3p and ITGA11 expression in the HGC-27 cell line by qRT-PCR assay after circRNA_100290 siRNA transfection was conducted. **E, F** CCK-8 assays were used to evaluate the effects of circRNA_100290 on the proliferation of AGS and HGC27 cells. Knocking down circRNA_100290 expression significantly inhibited GC cell viability. ***P < 0.001. **G, I** Colony formation assays were performed to evaluate the effects of circRNA_100290 on the colony-formation abilities of AGS and HGC27 cells. **H, J** The statistical analysis of the graphs showed that decreasing circRNA_100290 expression suppressed the AGS and HGC27 cell colony formation ability (*P < 0.05). **K, M** Flow cytometry was used to analyse the effects of circRNA_100290 on AGS and HGC27 cell cycle progression. **L, N** The statistical analysis of the graphs demonstrated that decreasing circRNA_100290 expression increased the percentage of AGS and HGC27 cells in the G0/G1 phase compared with the NC control groups (*P < 0.05)

The expression levels of circRNA_100290 and miR-29b-3p were assessed in four GC cell lines and GES-1. The expression of circRNA_100290 was upregulated in AGS, BGC-823, and HGC-27 cells compared with GES-1 cells, while miR-29b-3p was correspondingly downregulated (Fig. 3A, B). The AGS and HGC-27 cell lines were chosen for further knockdown of circRNA_100290 as a higher circRNA_100290 expression. After knocking down circRNA_100290, the expression of miR-29b-3p and ITGA11 presented increasing and decreasing trends, respectively, in both cell lines (Fig. 3C, D).

The CCK-8 assay was performed to compare the viability of GC cells. For five consecutive days, the absorbance value in si-circRNA_100290 AGS and HGC-27 cells was found to be obviously lower than that in the control groups, indicating that knocking down circRNA_100290 inhibited GC cell proliferation (Fig. 3E, F). The results of the cell colony formation assay demonstrated that the colony numbers in si-circRNA_100290 AGS and HGC-27 cells were lower than those in the control groups (Fig. 3G–J). Reduced circRNA_100290 expression suppressed the colony formation ability of GC cells. The cell cycle distribution was detected by flow cytometry. Decreased circRNA_100290 expression induced an increased percentage of cells in G0/G1 phase and downregulated the ratio of cells in S and G2/M phases in si-circRNA_100290 AGS and HGC-27 cells (Fig. 3K–N). The results suggested that knocking down circRNA_100290 might induce G0/G1 arrest and inhibit GC cell proliferation.

### CircRNA_100290 accelerated GC cell migration and invasion by regulating EMT

**Fig. 4:**
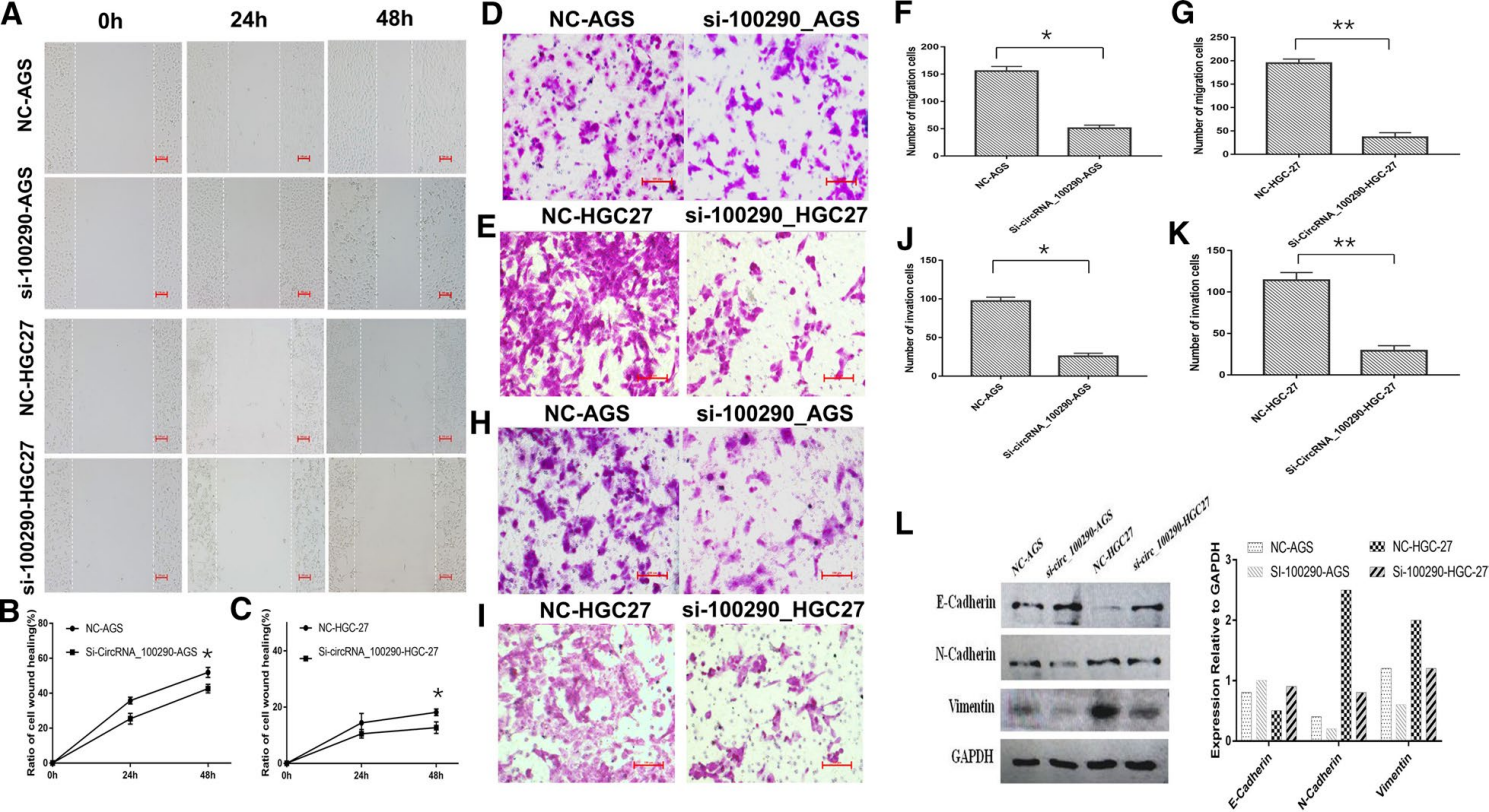
Decreased circRNA_100290 expression suppresses GC cell migration and invasion abilities and impedes EMT. **A** A wound healing assay was performed to evaluate the effects of circRNA_100290 on the migration abilities of AGS and HGC27 cells at 0 h, 24 h, and 48 h. **B**, **C** The statistical analysis of the graphs showed that knocking down circRNA_100290 obviously inhibited AGS and HGC27 cell wound healing (*P < 0.05). **D, E** Transwell migration assays were performed to evaluate the effects of circRNA_100290 on the migration abilities of AGS and HGC27 cells. **F, G** The statistical analysis of the graphs showed that decreased circRNA_100290 expression induced fewer AGS and HGC27 cells to migrate into the lower chamber compared with the control groups (*P < 0.05, ** P < 0.01). **H, I** Transwell invasion assays were performed to evaluate the effects of circRNA_100290 on the invasion abilities of AGS and HGC27 cells. **J, K** The statistical analysis of the graphs showed that reduced circRNA_100290 expression led fewer AGS and HGC27 cells invading the lower chamber compared with the control groups (*P < 0.05, ** P < 0.01). **L** Western blot assays were performed to evaluate the expression of EMT-related proteins, including E-cadherin, N-cadherin and Vimentin

Cell wound healing and Transwell migration assays were performed to measure the effect of circRNA_100290 on cell migration and invasion abilities. Cell wound healing assays demonstrated that the wound healing rate was significantly lower in the si-circRNA_100290 AGS and HGC-27 groups than in the control groups (Fig. 4A). The statistical diagrams of the AGS and HGC-27 groups showed that the most significant difference between the experimental and control groups was observed after 48 h (Fig. 4B, C). Transwell migration assays showed impaired migration ability in si-circRNA_100290 AGS and HGC-27 cells. The number of GC cells migrating into the lower chamber was lower in the si-circRNA_100290 groups than in the control group (Fig. 4D–G). The results of the Transwell invasion assay also revealed that the reduced expression of circRNA_100290 damaged the cell invasion ability. The number of AGS and HGC-27 cells invading the lower chamber was markedly reduced after knocking down circRNA_100290 (Fig. 4H–K). Moreover, Western blot assays demonstrated that knocking down circRNA_100290 increased the expression of E-cadherin and reduced the expression of N-cadherin and Vimentin (Fig. 4L).

### EIF4A3 could bind the flanking region of circRNA_100290 and inhibit circRNA_100290 expression in GC

**Fig. 5:**
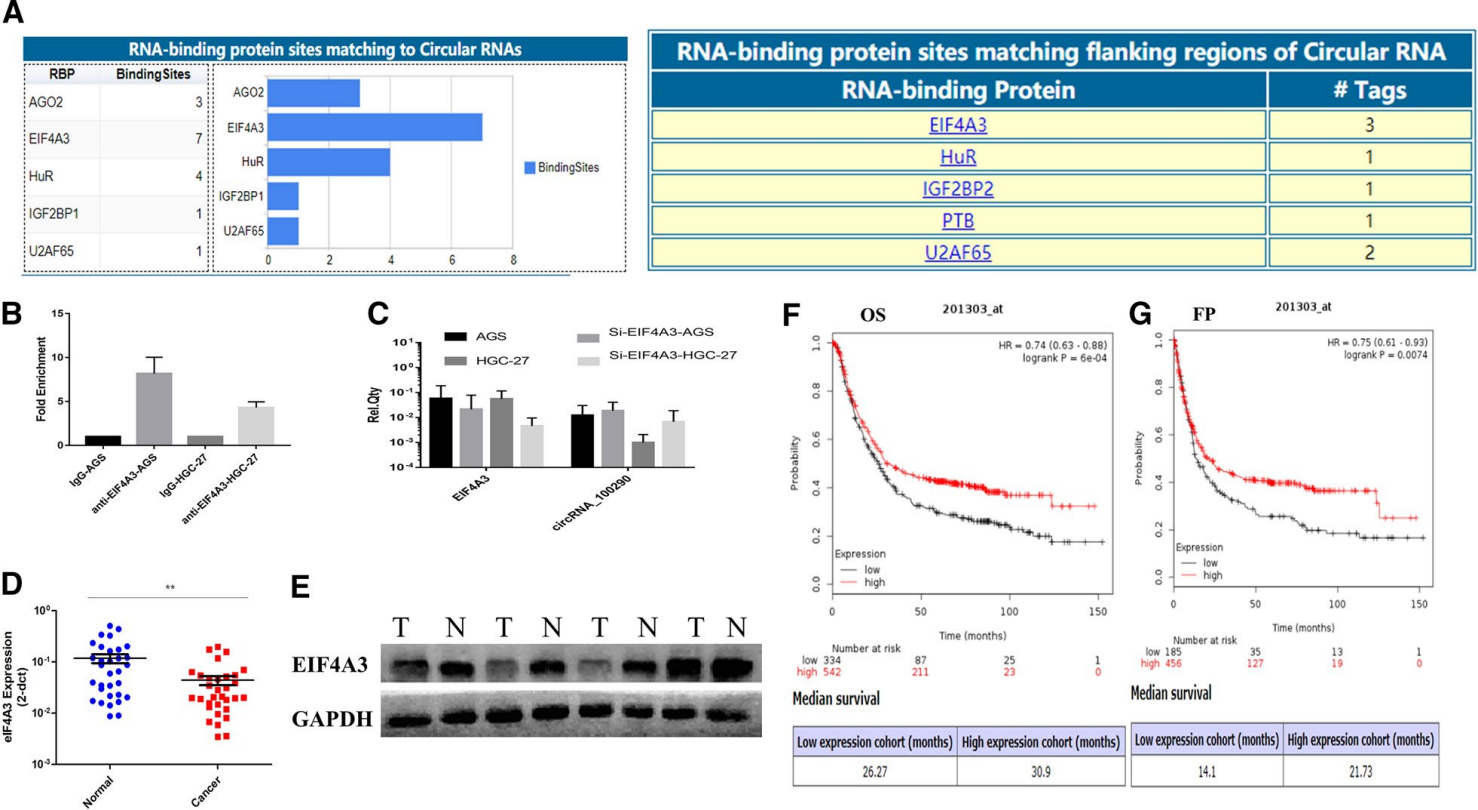
Transcriptional regulation of circRNA_100290 by EIF4A3 in GC. **A** Prediction of circRNA_100290-related RBPs by using CircInteractome software. **B** RIP assay was performed to determine fold enrichment of EIF4A3 on the flanking region of circRNA_100290. **C** Detection of circRNA_100290 by qRT-PCR after knocking down EIF4A3 in AGS and HGC-27 cells. **D** Detection of EIF4A3 expression in 31 GC tissues and paired normal tissues was conducted by qRT-PCR assay. Decreased expression of EIF4A3 was observed in GC tissues. The data are presented as the mean ± SEM. ** P < 0.01. **E** Western blot assay was performed to determine EIF4A3 expression in 31 GC tissues and paired normal tissues. **F, G** Analysis of ITGA11 in predicting of OS and FP in GC patients from the Kaplan–Meier plotter website

To determine the molecular mechanism underlying the regulation of circRNA_100290 expression, CircInteractome, a bioinformatics tool, was used to predict circRNA_100290-related RBPs. As shown in Fig. 5A, EIF4A3 had the most potential binding sites matched with circRNA_100290 and its flanking regions compared with other RBPs. Therefore, EIF4A3 was chosen for further study. RIP assays were performed to assess the binding between EIF4A3 and flanking regions of circRNA_100290 in AGS and HGC-27 cells. The RIP-qPCR results showed 8.18- and 4.31-fold enrichment of the flanking site of circRNA_100290 in AGS and HGC-27 cells, respectively (Fig. 5B). In addition, after the knockdown of EIF4A3, an increased level of circRNA_100290 was observed in both cell lines (Fig. 5C).

### Expression of EIF4A3 was decreased in GC, and low EIF4A3 expression predicted poor prognosis in patients with GC

qRT-PCR and Western blot assays were performed to assess the expression of EIF4A3 in 31 matched GC tissues. The results showed that the expression of EIF4A3 at both the mRNA and protein levels was significantly downregulated in GC (Fig. 5D, E). In addition, the prognostic value of the expression of EIF4A3 in 876 patients with GC from the GEO database was evaluated by using the Kaplan–Meier plotter website. The results showed that low EIF4A3 expression was associated with worse overall survival (OS) and first progression (FP). As shown in Fig. 5F, G, the median OS in the low-EIF4A3 expression group was 26.27 months, which was shorter than that in the high-EIF4A3 expression group (30.9 months). Comparably, the median FP in the low-EIF4A3 expression group was 14.1 months shorter than that in the high-EIF4A3 expression group (21.73 months). Subsequently, clinicopathological analysis was conducted to explore the role EIF4A3 plays in GC by using the GSE62254 dataset [27], which contains 300 GC patients from the GEO database. The results revealed that the expression of EIF4A3 in GC was closely related to sex, age, Lauren’s type, invasion depth, TNM staging, and ACRG genotyping (Table 3, P < 0.05). The data above suggested that EIF4A3 might serve as a suppressor in GC, and reduced expression of EIF4A3 predicted a worse prognosis of patients with GC.

**Table 3:** Relevance analysis between expression of EIF4A3 and clinicopathological characteristics in GC patients from GEO database^a^

Characteristics	N = 300	Expression of EIF4A3		χ2	*P*
		Low	High		
Gender				5.513	**0.019**
Male	199	20 (10.1%)	179		
Female	101	20 (19.8%)	81		
Age (years)				7.887	**0.005**
≤ 63	148	28 (18.9%)	120		
> 63	152	12 (7.9%)	140		
Lauren’s types				22.73	**0.000**
Intestinal	146	7 (4.8%)	139		
Diffuse	134	32 (23.9%)	102		
Mixed	17	1 (5.9%)	16		
Depth of invasion				8.897	**0.012**
T2	188	17 (9%)	171		
T3	91	20 (22%)	71		
T4	21	3 (14.3%)	18		
Ln metastasis				1.114	0.291
N0	38	3 (7.9%)	35		
N1 ~ N3	262	37 (15.3%)	225		
Distant Metastasis				0.902	0.342
M0	273	38 (13.9%)	235		
M1	27	2 (7.4%)	25		
TNM Staging				8.916	**0.030**
I	31	1 (3.2%)	30		
II	97	12 (12.4%)	85		
III	95	20 (21.1%)	75		
IV	77	7 (9.1%)	70		
ACRG’s mol. types				34.989	**0.000**
MSI	68	1 (1.5%)	67		
MSS/TP53^−^	107	8 (7.5%)	99		
MSS/TP53^+^	79	14 (17.7%)	65		
MSS/EMT	46	17 (37%)	29		

*Ln.* Lymph node, *ACRG* Asian Cancer Research Group, *mol.* Molecular, *MSI* microsatellite instability, *MSS* Microsatellite stability, *TP53*^*−*^ TP53 inactive, *TP53*^*+*^ TP53 active, *EMT* epithelial-to-mesenchymal transition

^a^GEO accession number: GSE62254

## Discussion

CircRNAs, a new class of noncoding RNAs (ncRNAs), have gradually gained attention. It has been reported that circRNAs that originate from exons and are located mainly in the cytoplasm usually function as miRNA ‘sponges’ [6]. CircRNAs also participate in transcriptional or post-transcriptional regulation [28, 29]. Moreover, several circRNAs containing internal ribosome entry sites could be translated into peptides [30]. However, the known circRNAs and their regulatory mechanism in GC have not been thoroughly elucidated. CircRNA_100290, one of the recently discovered circRNAs, is located on chromosome 1, and its parental gene is SLC30A7. A recent study reported that circRNA_100290 was abnormally highly expressed in colorectal cancer and promoted the proliferation of colorectal cells [15]. In the present study, circRNA_100290 was significantly upregulated in GC and was closely related to invasion depth, lymph node metastasis, and TNM stage. Functionally, silencing circRNA_100290 in AGS and HGC-27 cells significantly inhibited cell viability, colony formation, migration, and invasion ability and induced G0/G1 phase arrest in vitro. These results suggested that circRNA_100290 might serve as an oncogene in GC.

CircRNAs might function as miRNA sponges by binding to miRNAs and regulating downstream target genes, and this mechanism is also known as a competing endogenous RNA regulatory mechanism. In our study, the expression of miR-29b-3p was found to be decreased in GC, and this trend was validated by analysing data from the EBI database. MiR-29b-3p is a member of the miR-29 family, and decreased expression of miR-29 family members has been reported in various tumours, including lung cancer, oesophageal cancer, hepatocellular carcinoma, and so on [31–33]. The bioinformatics prediction and correlation analysis indicated that miR-29b-3p might share complementary binding sites with circRNA_100290, which was confirmed by the dual-luciferase reporter assay, suggesting that circRNA_100290 could function as a sponge for miR-29b-3p. Taken together, our results suggested that miR-29b-3p acted as a tumour suppressor and interacted with circRNA_100290 by sponging GC.

ITGA11, a candidate target of miR-29b-3p, was further studied. ITGA11 is a member of the integrin family. It is upregulated in various tumours, such as lung cancer, breast cancer, and meningeal glioma [34–36]. It was reported that increased ITGA11 expression in cancer stroma was correlated with a poor clinical outcome in patients with non-small-cell lung cancer (NSCLC). Mechanically, over-expressed ITGA11 promoted the cancer-associated fibroblasts (CAF) migration via ERK1/2 signalling pathway in NSCLC [37]. In breast cancer, it was reported that ITGA11 promoted CAF invasive activity by interacting with PDGFRβ and promoting its downstream JNK activation, leading to the production of tenascin C, a pro-invasive matricellular protein [38]. However, the role of ITGA11 in GC has not been reported thus far. The present study reported elevated expression of ITGA11 in GC, which was reversed by the expression of miR-29b-3p. Pathological factor analysis and survival analysis indicated that high expression of ITGA11 predicted a worse prognosis of GC patients. ITGA11 might serve as an oncogene in GC. Furthermore, silencing circRNA_100290 in AGS and HGC-27 cells led to increased miR-29b-3p expression and diminished ITGA11 expression, suggesting that circRNA_100290 could sponge miR-29b-3p to increase the expression of ITGA11, thereby promoting GC cell proliferation, migration and invasion.

Growing evidence demonstrates that circRNAs usually regulate tumour progression and metastasis by affecting EMT [39, 40]. In our study, knocking down circRNA_100290 induced the altered expression of several EMT markers, which was accompanied by the release of miR-29b-3p and inhibition of ITGA11. Previous studies have reported the involvement of miR-29b family members in EMT [41]. In addition, a report revealed that miR-29b could inhibit EMT and metastasis by targeting a network of prometastatic drivers involved in angiogenesis, collagen remodelling, and proteolysis [42]. Shin et al. reported that exogenous miR-29b mediated an anticancer effect by impeding the activation of ITGA11 [36]. In our study, by using PPI and GO analyses, ITGA11 was found to have a close association with EMT-related proteins such as TLN1 [43], FLNA [44], SPP1 [45], SPARC [46] etc., and could be involved in EMT. These results reflected that circRNA_100290 promoted EMT mainly via the miR-29b-3p/ITGA11 axis.

CircRNAs are formed via back-splicing regulated by RNA splicing factors or RBPs [47, 48]. In this study, among the RBPs, EIF4A3 was found to have the most predicted binding sites with both flanking regions and with circRNA_100290 itself, and the RIP assay confirmed the direct interaction between the flanking regions of circRNA_100290 and EIF4A3. As a member of the DEAD-box protein family, EIF4A3 is located mainly in the nucleus and is a part of the exon junction complex necessary for nonsense-mediated mRNA decay [49]. In addition, EIF4A3 is also involved in various biological processes, including mRNA translation initiation and RNA splicing [40, 51]. It was thought that EIF4A3 was probably involved in the transcriptional regulation of circRNA_100290. Wang et al. reported that EIF4A3 could induce circMMP9 cyclization and increase the expression of circMMP9 in GBM by binding to the MMP9 mRNA transcript [52]. Inconsistent with this finding, we found that silencing EIF4A3 led to elevated circRNA_100290 expression, indicating that EIF4A3 could inhibit the formation of circRNA_100290. The mechanisms by which circRNAs are regulated are complicated. Inverted Alu repeats could promote circRNA formation by facilitating the back-splicing of pre-mRNAs, while DHX9, an RBP, bound specifically to inverted-repeat Alu elements, thereby inhibiting the formation of circRNAs. The loss of DHX9 led to an increase in the number of circular RNAs [53]. In addition, Ivanov et al. reported that ADAR1 could decrease the expression of circRNA by competitively binding with reverse complementary sequences that are highly enriched in intron bracketing circRNAs [54]. EIF4A3 might inhibit the formation of circRNA_100290 by binding the flanking regions of circRNA_100290. These studies suggested that the role of EIF4A3 in cyclization of different circRNAs is distinct, and the underlying mechanism remains to be further explored.

Additionally, we found that the expression of EIF4A3 was downregulated in GC. Moreover, low EIF4A3 expression was found to predict a worse prognosis and was closely related to Lauren’s type, invasion depth, and TNM staging based on analyses of pathological factors in GSE62254, a dataset from the GEO database, which includes 300 GC patients. By comprehensive sequencing, 300 GC cases were classified into four subtypes, including MSI, MSS/EMT, MSS/TP53 + and MSS/TP53-, according to the ACRG molecular type [55]. Interestingly, the EIF4A3 high-expression group had the lowest percentage of MSS/EMT subtypes of ACRG genotyping. A previous study reported that GC patients with the MSS/EMT subtype, especially those with peritoneal dissemination, had the worst OS and the highest rate of recurrence [56]. The results indicated that EIF4A3 functioned as a tumour suppressor and negative regulator of circRNA_100290 in GC.

## Conclusion

In conclusion, our study reveals that the expression of circRNA_100290 was upregulated in GC. Furthermore, the knockdown of circRNA_100290 significantly inhibited GC cell proliferation, migration, and invasion in vitro. The potential mechanism could be that circRNA_100290 regulated EMT by targeting miR-29b-3p/ITGA11. In addition, EIF4A3 could serve as a negative regulator of circRNA_100290 and a tumour suppressor in GC. CircRNA_100290 might serve as a potential biomarker and an effective target for GC diagnosis and therapy.

## Data Availability

Not applicable.

## Abbreviations

GC: Gastric cancer
CircRNA: Circular RNA
miRNA: MicroRNA
MRE: MiRNA response element
EMT: Epithelial-mesenchymal transition
RBP: RNA-binding proteins
RIP: RNA Immunoprecipitation
OS: Overall survival
FP: First progression
ACRG: Asian Cancer Research Group
